# Chinese Herbal Compounds for the Prevention and Treatment of Atherosclerosis: Experimental Evidence and Mechanisms

**DOI:** 10.1155/2015/752610

**Published:** 2015-05-18

**Authors:** Qing Liu, Jianping Li, Adam Hartstone-Rose, Jing Wang, Jiqiang Li, Joseph S. Janicki, Daping Fan

**Affiliations:** ^1^Department of Cell Biology and Anatomy, University of South Carolina School of Medicine, 6439 Garners Ferry Road, Columbia, SC 29208, USA; ^2^Guangdong Provincial Hospital of Chinese Medicine, The Second Clinical School of Medicine, Guangzhou University of Chinese Medicine, Guangzhou 510405, China

## Abstract

Atherosclerosis is a leading cause of disability and death worldwide. Research into the disease has led to many compelling hypotheses regarding the pathophysiology of atherosclerotic lesion formation and the resulting complications such as myocardial infarction and stroke. Herbal medicine has been widely used in China as well as other Asian countries for the treatment of cardiovascular diseases for hundreds of years; however, the mechanisms of action of Chinese herbal medicine in the prevention and treatment of atherosclerosis have not been well studied. In this review, we briefly describe the mechanisms of atherogenesis and then summarize the research that has been performed in recent years regarding the effectiveness and mechanisms of antiatherogenic Chinese herbal compounds in an attempt to build a bridge between traditional Chinese medicine and cellular and molecular cardiovascular medicine.

## 1. Introduction

Atherosclerosis is a disease of the arterial wall that occurs at susceptible sites in major arteries. It is initiated by endothelial injury and subsequent lipid retention and oxidation in the intima which then provokes chronic inflammation and ultimately causes stenosis or thrombosis [1]. During this progression, residential arterial wall cells including endothelial cells (ECs) and vascular smooth muscle cells (VSMCs), as well as circulating leukocytes, especially monocytes/macrophages, are mainly involved. Atherosclerotic lesions can cause stenosis with potentially lethal distal ischemia or, if ruptured, can trigger thrombotic occlusion of major arteries to the heart, brain, legs, and other organs [2]. A variety of risk factors may intensify or provoke atherosclerosis through their effects on endothelial function, low-density lipoprotein (LDL) concentration and modification, and vascular wall inflammation. These risk factors include hypertension, smoking, diabetes mellitus, obesity, and bacterial infection [3].

Traditional Chinese medicine (TCM), especially herbal medicine, has been used for the treatment of cardiovascular diseases for hundreds of years as documented in* Inner Canon of Yellow Emperor* and* Synopsis of Golden Chamber*. Also, the effectiveness of several extracts derived from Chinese herbs has been evaluated in recent years. However, the cellular and molecular details regarding the underlying efficacious mechanisms of Chinese herbal medicine in treating atherosclerosis have just begun to be understood. Therefore, the purpose of this review is to first provide a brief description of the mechanisms of atherogenesis and then to summarize the recent research results regarding the effectiveness and mechanisms of antiatherogenic Chinese herbal compounds.

## 2. Mechanisms of Atherogenesis

**Figure 1:**
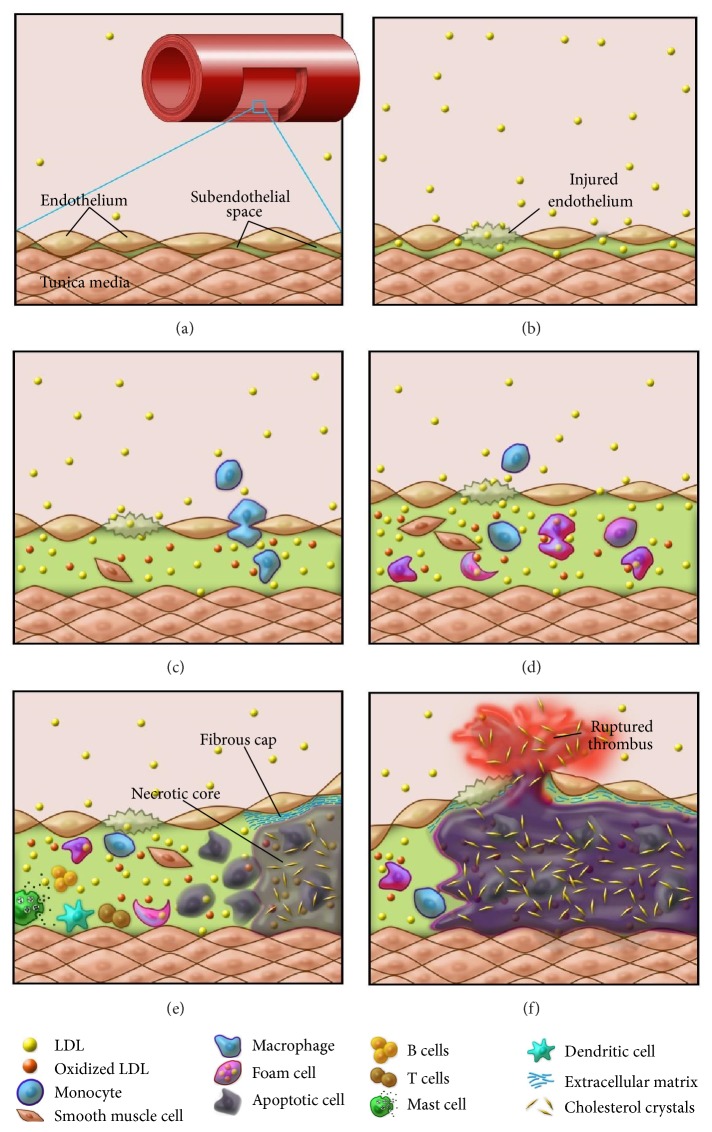
A schematic drawing depicting the formation of atherosclerotic plaques. (a) In the wall of a normal artery, there is a very small subendothelial space in the intima between the endothelium and the smooth muscle cell layer in tunica media. (b) Hyperlipidemia and endothelial injury lead to the infiltration of LDL particles into the subendothelial space. (c) A large number of LDL particles are retained and subsequently oxidized in the subendothelial space, followed by monocyte infiltration (from lumen) and smooth muscle cell migration (from tunica media). (d) Monocytes and smooth muscle cells differentiate into macrophages, which engulf LDL and turn into foam cells, and are activated by oxidized LDL. SMCs are also activated, proliferate, and transform into lipid-laden foam cells. (e) Macrophage and smooth muscle foam cells undergo apoptosis; unbalanced apoptosis/efferocytosis results in necrotic core formation and unresolved inflammation. Other immune cell types also participate in the arterial wall inflammation. (f) Erosion of the fibrous cap caused by the matrix degrading enzymes secreted by the macrophages leads to unstable plaques, which eventually rupture and result in thrombus formation and adverse clinical events.

Atherogenesis is an inflammatory process, initiated by the retention of lipids in the subendothelial space of the vascular wall and encompasses a complex interaction among the modified lipoproteins, residential vascular cells, and immune system [4]. The schematic in Figure 1 depicts the main steps of atherogenesis. In the following section, the main elements involved in the pathogenesis of atherosclerosis will be briefly described.

### 2.1. Hyperlipidemia

Dyslipidemia is one of the main risk factors leading to atherosclerosis [5]. The lipid hypothesis of atherogenesis states that abnormally elevated levels of plasma LDL and low levels of plasma high density lipoproteins (HDL) are the primary causes of atherosclerosis [6–8]. This hypothesis has been strongly supported by the success of statin drug therapy, which has significantly reduced coronary artery disease mortality through lowering plasma LDL levels during the past 40 years [9]. However, the HDL wing of the hypothesis remains to be confirmed by successful HDL-targeting approaches. A common mechanism through which hyperlipidemia causes atherosclerosis involves the accumulation of cholesteryl esters in macrophages of the arterial wall [10].

### 2.2. Endothelial Injury

The response-to-injury hypothesis of atherogenesis states that endothelial injury triggers subsequent interactions among all of the cells found in the atherosclerosis lesions [11]. Injured endothelium allows lipoproteins to migrate into subendothelial space. This, together with the discovery of adhesion molecules expressed by endothelial cells (e.g., vascular cell adhesion molecule-1), provides important insight into the initiation of atherosclerotic lesions [12]. That is, increased expression of adhesion molecules favors monocyte adhesion and penetration, which results in accumulation of macrophages within the subendothelial space where they encounter lipoprotein particles [13].

### 2.3. LDL Subendothelial Retention and Oxidation

Subendothelial retention of lipoproteins is a key early step in atherosclerosis, provoking a cascade of adverse events to the pathogenic response [14]. High levels of plasma lipids, particularly LDL and very-low density lipoproteins (VLDL), are among the pathophysiologic stimuli that induce endothelial dysfunction. Retention and modification of apolipoprotein B (apoB) containing lipoproteins, LDL, intermediate density lipoprotein (IDL), and lipoprotein (a) [Lp(a)] in the arterial intima extracellular matrix (ECM) represent early events of plaque development, which is referred to as the “response-to-retention” hypothesis [15].

The oxidation hypothesis of atherosclerosis suggests that an early event in the development of atherosclerosis is an oxidative modification of LDL that significantly increases its uptake into the arterial intima [16, 17]. Moreover, lipid overload may increase lipopolysaccharide (LPS) circulating levels and oxidative stress. In particular, the oxidation of lipoproteins that results from an imbalance of the pro- and antioxidant equilibrium is involved in the pathologic process of atherosclerotic alterations of cellular function. Lipid oxidation, induced by leukocyte-derived reactive oxygen species, not only promotes the growth and migration of smooth muscle cells, monocytes/macrophages, and fibroblasts, but also amplifies foam cell formation through oxidized LDL (oxLDL) formation and uptake [18].

### 2.4. Monocyte Migration and Activation

The overexpression of inducible adhesion molecules results in the adherence of mononuclear cells to the endothelial surface whereupon they receive chemoattractant signals that beckon them to enter the intima. With regard to the mechanisms that mediate monocyte-derived macrophage maturation, it has been reported that macrophage colony-stimulating factor (M-CSF) induces scavenger receptors and promotes the proliferation of monocytes in early atherosclerotic lesions [19]. Macrophages also contribute to the thrombotic complications of atherosclerosis in pivotal ways. These phagocytes furnish the bulk of the enzymes (i.e., matrix metalloproteinases, MMPs) that catabolize collagen, a key constituent of the fibrous cap of the plaque, which when activated predisposes the plaque to rupture [20].

### 2.5. Vascular Smooth Muscle Cell (VSMC) Migration and Proliferation

In response to atherogenic stimuli, VSMCs undergo a phenotypic switch from contractile phenotype to synthetic and inflammatory phenotype; the inflammatory VSMCs migrate into intima and proliferate, contributing to the atherogenesis [21, 22]. VSMCs are the major producers of ECM within the vessel wall [23] and can modify the type of matrix proteins produced. In turn, the type of matrix present can affect the lipid content of the developing plaques and the proliferative index of the cells that are adherent to them. Like endothelial cells, VSMCs can also express a variety of adhesion molecules such as vascular cell adhesion molecule-1 (VCAM-1) and intercellular adhesion molecule-1 (ICAM-1) to which monocytes and lymphocytes can adhere and migrate into the vessel wall [24]. Like macrophages, VSMCs can also express a variety of receptors for lipid uptake and can form foam-like cells, thereby participating in the early accumulation of plaque lipid [25].

### 2.6. Foam Cell Formation

Foam cells mainly arise from mononuclear phagocytes, although smooth muscle and endothelial cells can also become engorged with lipids. Within the plaque, the mononuclear phagocytes express scavenger receptors (SRs), including CD36, SR-A, and SR-BI. These scavenger receptors mediate the engulfment of modified LDL particles that contribute to macrophage foam cell formation [26]. Other receptors for native lipoprotein particles, including LDLR, VLDLR, and LRP1, also contribute to foam cell formation. As mentioned above, VSMCs, which acquire a synthetic and inflammatory phenotype in the plaque, can also take up lipoproteins and transform into foam cells [27]. Death of foam cells leads to formation of a necrotic core, which serves as a depot for cellular debris and lipids [28].

### 2.7. Apoptosis and Efferocytosis and Unresolved Inflammation

As atherosclerotic lesions evolve, both the macrophage-derived and smooth muscle-derived foam cells can undergo programmed cell death or apoptosis [29]. The death of foam cells may not be a random event or the result of bursting like an overinflated balloon due to lipid overload. Rather, it may be due in part to gradients of concentration of factors such as macrophage colony-stimulating factor (M-CSF) required for survival of human monocytes [20]. However, some apoptotic cells may not disappear from the atherosclerotic lesions but instead accumulate in a “mummified” state [20]. The elegant studies of Ira Tabas have elaborated upon this concept of impaired clearance, or “efferocytosis,” of apoptotic cells in plaques, which leads to unresolved inflammation [30]. The apoptotic foam cells that escape efferocytosis release their lipid content to the extracellular space and contribute to lipid core formation.

Over the last dozen years, appreciation of the role of inflammation in atherosclerosis has burgeoned. Intralesional or extralesional inflammation may hasten atheroma evolution and precipitate acute events. Circulating acute-phase reactants elicited by inflammation not only may serve as a biomarker for increased risk of vascular events, but also in some cases may contribute to their pathogenesis [31]. Advances stemming from basic research have established a fundamental role for inflammation in mediating all stages of this disease from initiation through progression and, ultimately, to the thrombotic complications of atherosclerosis.

The basic science of inflammatory biology applied to atherosclerosis has provided considerable insight into the mechanisms underlying the recruitment of leukocytes. Early after the initiation of atherogenesis, arterial endothelial cells begin to express on their surface selective adhesion molecules that bind various classes of leukocytes [12]. In particular, VCAM-1 binds precisely the types of leukocytes involved in early atheroma, the monocyte and T lymphocyte. Not only does VCAM-1 expression increase on endothelial cells overlying nascent atheroma, but defective VCAM-1 shows interrupted lesion development [32]. Once adhered to the endothelium, leukocytes penetrate into the intima in response to chemoattractant molecules. For example, monocyte chemoattractant protein-1 (MCP-1) appears responsible for the direct migration of monocytes into the intima at sites of lesion formation [33]. Once resident in the arterial wall, the blood-derived inflammatory cells participate in and perpetuate a local inflammatory response. The macrophages express scavenger receptors for modified lipoproteins, permitting them to ingest lipid and become foam cells. In addition to MCP-1, macrophage colony-stimulating factor (M-CSF) contributes to the differentiation of the blood monocyte into the macrophage foam cell [34]. T cells likewise encounter signals that cause them to elaborate inflammatory cytokines such as tumor necrosis factor-*α* (TNF-*α*) that in turn can stimulate macrophages as well as vascular endothelial cells and SMCs [35]. As this inflammatory process continues, the activated leukocytes and intrinsic arterial cells can release fibrogenic mediators including a variety of peptide growth factors that can promote replication of SMCs and contribute to elaboration by these cells of a dense ECM characteristic of a more advanced atherosclerosis lesion. Inflammatory processes not only promote initiation and evolution of atheroma, but also contribute decisively to precipitating the acute thrombotic complications of atheroma [3]. The activated macrophages abundant in atheroma can produce proteolytic enzymes capable of degrading the collagen that lends strength to the plaque's protective fibrous cap, rendering the cap thin, weak, and susceptible to rupture. Inflammatory mediators regulate tissue factor expression by plaque macrophages, demonstrating an essential link between arterial inflammation and thrombosis [36].

Both innate and adaptive immunity are involved in atherosclerosis. Inflammation per se can drive arterial hyperplasia, even in the absence of traditional risk factors [37]. Cytokines as inflammatory messengers provide a mechanism whereby risk factors for atherosclerosis can alter arterial biology. Inflammation regulates aspects of plaque biology that trigger the thrombotic complications of atherosclerosis [38]. Overall, inflammatory mediators participate in all phases of atherogenesis, from lesion initiation through progression and ultimately to the clinical complications of this disease. The fact that all types of immune cells have been found in atherosclerotic plaques indicates that all immune components may participate in atherogenesis. All of these factors form the basis of the “inflammatory hypothesis.”

## 3. Effects and Mechanisms of Chinese Herb Compounds in the Attenuation of Atherosclerosis

An early description of the clinical manifestations and treatment of atherosclerosis can be found in the classic traditional Chinese medicine book* Inner Canon of Yellow Emperor*, which was completed around 500 BC. In the theory of traditional Chinese medicine, atherosclerosis is usually referred to as “*MaiBi,*” a vascular problem that is caused by* Qi* stagnation,* Blood* stasis, and/or coagulated* Phlegm*, in which* Qi* stands for the energy,* Blood* stands for the material, and* Phlegm* stands for a kind of pathological product. For over two thousand years, atherosclerosis and its resulting heart disease have been treated with numerous herbal remedies. While somewhat effective, these herbal remedies have not been well studied using evidence-based approaches or using modern cellular and molecular techniques. Recently, however, investigations to examine the effects and mechanisms of single herbal compounds in the modulation of atherogenesis have occurred. A summary of these studies is presented in the following section wherein the compounds are discussed according to their site of activity.

### 3.1. Chinese Herbal Compounds with Endothelial Protective Activity (Table 1)

The study by Lee et al. demonstrated that pretreatment of human umbilical vein endothelial cells (HUVEC) with* Buddleja Officinalis* (BO, 1–10 microg/mL) for 18 hrs dose-dependently inhibited TNF-*α*-induced adhesion U937 monocytic cells as well as mRNA and protein expressions of VCAM-1 and ICAM-1. Pretreatment with BO also blocked TNF-*α*-induced reactive oxygen species (ROS) formation. Nuclear factor-kappa B (NF-kappa B) is required for the transcription of these adhesion molecule genes [43]. Wan et al. found that* Panax notoginseng saponins* (PNS), derived from the Chinese herb* Panax notoginseng*, dose-dependently inhibited monocyte adhesion to activated endothelium, as well as the expression of TNF-*α*-induced endothelial adhesion molecules, such as ICAM-1 and VCAM-1 [45]. Recent findings reported by Tian et al. indicated that* Resveratrol*, a compound derived from the Chinese herb* Rhizoma polygonum cuspidatum*, downregulated the increased expressions of vascular endothelial growth factor (VEGF) and kinase insert domain receptor (KDR or VEGF receptor-2) [39]. Results from Choi et al. showed that* extract from Cynanchum wilfordii* (ECW) treatment significantly decreased vascular inflammation through an inhibition of cellular adhesion molecules such as E-selectin, VCAM-1, and ICAM-1 as well as endothelin-1 (ET-1) expression [40].

### 3.2. Chinese Herbal Compounds That Lower Lipids and Antioxidation (Tables 2 and 3)

Zhang et al. [54] using a plasma lipid analysis approach found* Celastrus orbiculatus Thunb Extract* (COT), a compound derived from the Chinese herb* Celastrus orbiculatus Thunb*, to decrease total cholesterol (TC), non-high-density lipoprotein cholesterol (non-HDL-C), triglyceride (TG), apolipoprotein B100 (apoB100), and apolipoprotein E (apoE) levels and to increase the level of HDL cholesterol (HDL-C). Quantitative real-time PCR revealed that COT upregulated the mRNA abundance of LDL receptor (LDL-R), scavenger receptor class B type 1 (SR-B1), cholesterol 7*α*-hydroxylase A1 (CYP7A1), and 3-hydroxy-3-methyl-glutaryl-CoA reductase (HMGCR) [54]. Choi et al. reported that extract from the herb* Cynanchum wilfordii treatment* in HFCD-fed rats lessened LDL cholesterol and triglyceride levels and elevated HDL cholesterol [40]. Results from Subramaniam et al. indicated that the ethanolic fraction of the herb* T. arjuna* significantly decreased TC, LDL, and TG levels, increased HDL, and lessened the number of aortic atherosclerotic lesions [57]. Dinani et al. demonstrated the ability of the extract from the Chinese herb* Artemisia aucheri* to significantly reduce the levels of TC, LDL cholesterol, and TG and to increase HDL cholesterol [58].

Li et al. discovered that* Farrerol,* an extract from the Chinese herb* Rhododendron dauricum L.*, significantly inhibited the H_2_O_2_-induced loss of cell viability and enhanced superoxide dismutase (SOD) and glutathione peroxidase (GSH-Px) activities in EA.hy926 cells. In addition,* Farrerol* inhibited the H_2_O_2_-induced elevation in the levels of intracellular malondialdehyde (MDA) and reactive oxygen species (ROS) [63]. Chen et al. reported that treatment with* Salvianolic acid B* (Sal B), a main compound derived from the herb* Salvia miltiorrhiza* Bunge, suppressed ERK1/2 and JNK phosphorylation and attenuated the increase in prostaglandin E2 production and NADPH oxidase activity in LPS-treated human aortic smooth muscle cells (HASMCs), indicating that Sal B has antioxidant properties [71]. Jia et al. showed that Tanshinone IIA (TSN IIA), another main compound derived from the Chinese herb* Salvia Miltiorrhiza* Bunge, markedly inhibited the elevation of ROS evoked by H_2_O_2_. Real time RT-PCR and Western blotting analysis demonstrated the ability of TSN IIA to significantly decrease the H_2_O_2_-induced expression of proapoptotic proteins Bax and caspase-3 and to significantly increase the expression of antiapoptotic protein Bcl-2 in EA.hy926 cells [64].

Results from Xu et al. showed that the Lectin-like oxidized LDL (oxLDL) receptor-1 (LOX-1), a novel scavenger receptor highly expressed in human and experimental atherosclerotic lesions, is responsible for the uptake of oxLDL in vascular cells. oxLDL induced LOX-1 expression at the mRNA and protein levels, which was abrogated by the addition of Tanshinone IIA or a widely used inhibitor of NF-*κ*B, suggesting the involvement of NF-*κ*B [65]. Hung et al. described that a low dose (0.015 mg/mL) of* S. miltiorrhiza* aqueous extract (SMAE), derived from the Chinese herb* Salvia miltiorrhiza* Bunge, significantly inhibited the growth of a rat smooth muscle cell line (A10) under Hcy stimulation, and the intracellular ROS concentration decreased after SMAE treatment in terms of reducing p47 (phox) translocation and increasing catalase activity. The signaling profile suggests that SMAE inhibited Hcy-induced A10 cell growth via the PKC/MAPK-dependent pathway [68].

### 3.3. Chinese Herbal Compounds That Suppress Monocyte Migration and Activation (Table 4)

Within plaque formation, activated endothelial cells increase the expression of adhesion molecules and inflammatory genes and circulating monocytes migrate into subendothelial space and differentiate into macrophages. In support of this concept, Chen et al. found that extract from* Ginkgo biloba*, a Chinese herb with antioxidant activity, could significantly suppress inflammatory cytokine-stimulated endothelial adhesiveness to human monocytic cells by attenuating intracellular ROS formation, redox-sensitive transcription factor activation, and VCAM-1 as well as ICAM-1 expression in human aortic endothelial cells [46]. Wan et al. found that* Panax notoginseng saponins* (PNS) dose-dependently inhibited monocyte adhesion on activated endothelium, as well as the expression of TNF-*α*-induced endothelial adhesion molecules, such as ICAM-1 and VCAM-1 [45]. According to the report by Park,* Prunella vulgaris ethanol* extract inhibited adhesion of monocyte/macrophage-like THP-1 cells to the activated HASMCs [91]. The role of* Curcumin*, derived from the Chinese herb Curcuma longa, was shown by Wang et al. to have a sonodynamic effect on THP-1-derived macrophages and, therefore, to be a promising treatment for atherosclerosis [92]. Finally, Duan et al. identified* Phyllanthus emblica extract* as being able to prevent ECV-304 cells from adhering to monocytes [79].

### 3.4. Chinese Herbal Compounds That Suppress VSMC Migration and Proliferation (Table 5)

Several lines of evidence exist to indicate the effectiveness of Chinese herbs on VSMC migration and proliferation. Moon et al. observed that* Protocatechuic aldehyde* (PCA), a compound derived from the Chinese herb* Salvia miltiorrhiza Bunge*, significantly attenuated PDGF-induced VSMC proliferation and migration at a pharmacologically relevant concentration (100 *μ*M). On a molecular level, they observed downregulation of the phosphatidylinositol 3-kinase (PI3 K)/Akt and the mitogen-activated protein kinase (MAPK) pathways, both of which are known to regulate key enzymes associated with migration and proliferation. Moreover, they found that PCA arrested the S-phase of the VSMC cell cycle and suppressed cyclin D2 expression [93]. Results from Kim et al. indicated that* Corynoxeine*, derived from the Chinese herb* Hook of Uncaria rhynchophylla*, significantly inhibited the PDGF-BB-induced DNA synthesis of VSMCs in a concentration-dependent manner without causing any cytotoxicity. Preincubation of VSMCs with corynoxeine significantly inhibited PDGF-BB-induced extracellular signal-regulated kinase 1/2 (ERK1/2) activation [95]. Liang et al. showed that* Berberine*, a compound from the Chinese herb* Coptis chinensis*, inhibited serum-stimulated rat aortic VSMC growth in a concentration-dependent manner. Berberine blocked injury-induced VSMC regrowth by inactivation of the ERK/Egr-1 signaling pathway thereby preventing the early signaling induced by injury in vitro [96].

### 3.5. Chinese Herb Compounds That Suppress Foam Cell Formation (Table 6)

In the studies reported by Yuan et al., the formation of foam cells was inhibited by* Panax notoginseng saponins* (PNS) via its ability to inhibit the phosphorylation of FAK on threonine 397 and the translocation of NF-*κ*B. Wang et al. discovered that TNF-*α* could enhance the activity of NF-kappa B in the foam cells, and this effect could be attenuated by* Astragalus polysaccharide* (APS), a compound derived from the Chinese herb* Astragalus membranaceus* [99]. In a study by Chen et al, large numbers of monocytes were found adherent to the luminal surface and a markedly thickened intima filled with many lipid laden foam cells was apparent. However when treated with* Scoparone*, a compound derived from the Chinese herb* Artemisia scoparia*, atherosclerosis was less advanced and the plasma cholesterol was lower [87]. Interestingly, Chen et al. reported that upon histopathological examination* Hibiscus sabdariffa Extract* (HSE) was noted to reduce foam cell formation and inhibit smooth muscle cell migration and calcification in the blood vessel of rabbits. These results clearly indicate that Chinese herb-derived extracts can be used to lower serum lipids and produce antiatherosclerotic activity [98].

### 3.6. Anti-Inflammatory Chinese Herb Compounds (Table 7)

Intralesional or extralesional inflammation may hasten atheroma evolution and precipitate acute adverse events. Hence, herb-associated treatment targeting inflammation is beneficial. From the findings of Jia et al., real time RT-PCR and Western blotting analysis revealed that* Tanshinone IIA* (TSN IIA) significantly decreased the expressions of the proapoptotic proteins Bax and caspase-3, significantly increased the expression of antiapoptotic protein Bcl-2, and resulted in the reduction of the Bax/Bcl-2 ratio in EA.hy926 cells induced by H_2_O_2_ [64]. Li et al. reported that* Farrerol* inhibited H_2_O_2_-induced elevation in the levels of intracellular malondialdehyde and ROS, as well as cell apoptosis [63]. Xing et al. found that LPS (15 *μ*g/mL) stimulation for 30 hr resulted in significant HUVEC apoptosis, as detected by Hoechst 33258 staining and Annexin V analysis and that* Protocatechuic aldehyde* (PCA, 0.25–1.0 mmol/L, 12 h) inhibited the apoptosis in a dose-dependent manner [41].

Recently, the research of Napagoda et al. indicated that the ethnopharmacological use of* Plectranthus zeylanicus extract* constituted an anti-inflammatory remedy [101]. Zhang et al. found that* Celastrus orbiculatus Thunb* (COT) lowered the levels of C-reactive protein (CRP), interleukin-6 (IL-6), and TNF-*α* in plasma [54]. Wang et al. discovered that* Artemisinin*, a compound derived from the Chinese herb* Artemisia annua*, inhibited the secretion and mRNA levels of TNF-*α*, interleukin (IL)-1*β*, and IL-6 in a dose-dependent manner in THP-1 human monocytes. They also found that the NF-*κ*B pathway may be involved in a decreased cytokine release [107]. Chen and Cheng reported that the extract from Chinese herb* Feverfew* effectively reduced LPS-mediated TNF-*α* and CCL2 (MCP-1) release by THP-1 cells [109].

## 4. Summary and Perspective

Herein, we have reviewed most of the Chinese herbal compounds recently reported to have antiatherogenic properties either in vitro or in vivo. Chinese herbal medicine has the potential to provide a major public health benefit by reducing morbidity and mortality secondary to cardiovascular disease. Recent experimental prevention and treatment studies using Chinese medicine clearly demonstrate the benefits of lowering LDL retention and LDL oxidant, protecting endothelium, inhibiting monocyte/macrophage/VSMC proliferation and migration, and preventing foam cell formation as well as the accompanying inflammation. While the promise of Chinese herb-derived compounds as effective therapies for atherosclerotic cardiovascular diseases has been indicated in the literature, the published studies have severe limitations and apparently more research is required. Firstly, most of the clinical studies are of limited value because of the small sample size and/or incomplete data and most experimental studies have focused mainly on single compounds extracted from Chinese herbs. Studies of Chinese decoctions or formulations are scarce, although decoction and formulations are the main forms of therapy in traditional Chinese medicine practice. Capitalization of the interactions between the different components and herbs is the essence of traditional Chinese medicine whereby herbs are combined to attenuate toxicity as well as to enhance efficacy. Secondly, like other therapies, Chinese herbs and the compounds derived from them are expected to have side effects. However, published in vivo studies seldom mention whether adverse effects occurred. In future studies, including animal studies and clinical studies, systemic and organ-specific side effects of Chinese herb medicine should be carefully examined. Thirdly, in modern medical practice, it is unlikely that Chinese herbal medicine will be used as a sole treatment for cardiovascular disease; instead, they will more likely be used in combination with other proven drugs. Therefore, the herb-drug interaction should be carefully evaluated in future studies where Chinese herbs or compounds are used in addition to traditional proven therapies. Fourth, atherosclerosis is a multiple-staged and multifaceted disease; most published studies are focused on examining the effects of Chinese herb medicine on one or only a few aspects of the disease. In future studies, a more systemic evaluation of the effects of Chinese herbal medicine on all aspects of atherosclerosis should be performed, including lipoprotein metabolism, endothelial injury, systemic and arterial local inflammation, as well as cell apoptosis and efferocytosis dynamics/balance in the plaques.

## Figures and Tables

**Table 1:** Chinese herbal compounds with endothelial protective activity.

Compound	Herb	Target or indicator	Type of study	Reference
Resveratrol	*Rhizoma polygonum cuspidatum *	cav-1, VEGF, KDR	In vitro	[39]
*Cynanchum wilfordii *	*Cynanchum wilfordii *	LDL, HDL, NO, E-selectin, VCAM-1, ICAM-1, ET-1	In vivo	[40]
Protocatechuic aldehyde	*Salvia miltiorrhiza* Bunge	Caspase-3, caspase-2, Bcl-2/Bax, cytochrome c, caspase-9, granzyme B	In vitro	[41]
Cryptotanshinone	*Salvia miltiorrhiza* Bunge	oxLDL, NO, ICAM-1, VCAM-1; monocyte adhesion	In vitro	[42]
Aqueous extract of *Buddleja officinalis *	*Buddleja officinalis *	VCAM-1, ICAM-1; ROS; NF-*κ*B	In vitro	[43]
*Tribulus terrestris* extract	*Tribulus terrestris *	TC, HDL, LDL, TG	In vivo	[44]
*Panax notoginseng *	*Panax notoginseng saponins *	ICAM-1 and VCAM-1	In vivo	[45]
*Ginkgo biloba* extract	*Ginkgo biloba *	VCAM-1, ICAM-1, E-selectin; ROS, RSTF	Both	[46, 47]
*Salvia miltiorrhiza *	*Salvia miltiorrhiza* Bunge	eNOS, NO, NADPH oxidase subunit Nox4	In vitro	[48]
Bisacurone	*Curcuma longa* Linne (Zingiberaceae)	VCAM-1, NF-*κ*B p65, Akt, PKC	In vitro	[49]
Magnolol	*Magnolia officinalis *	IL-6, STAT3, Tyr705 and Ser727, ICAM-1, IREs, monocyte adhesion, cyclin D1, MCP-1, NF-kB, VCAM-1	Both	[50, 51]
Aqueous extract of *Salvia miltiorrhiza *	*Salvia miltiorrhiza* Bunge	ICAM-1, VCAM-1, GSH, NF-*κ*B	In vitro	[52]
Salvianolic acid B	*Salvia miltiorrhiza* Bunge	ICAM-1, E-selectin, NF-*κ*B	In vitro	[53]

**Table 2:** Chinese herbal compounds that lower lipids.

Compound	Herb	Target or indicator	Type of study	Reference
*Celastrus orbiculatus* Thunb.	*Celastrus orbiculatus* Thunb.	TC, non-HDL, TG, apoB100, apoE, HDL; LDL receptor, SR-B1, CYP7A1, HMGCR, CRP, MDA	In vivo	[54]
Salvianolic acid B	*Salvia Miltiorrhiza* Bunge	mLDL, CD36	In vitro	[55]
*Cynanchum wilfordii *	*Cynanchum wilfordii *	LDL, HDL, NO; Akt,	In vivo	[56]
Ethanolic fraction of *T. arjuna *	*Terminalia arjuna *	LDL, TG, VLDL, HDL	In vivo	[57]
*Artemisia aucheri *	*Artemisia aucheri *	Total cholesterol, LDL cholesterol, triglycerides, HDL cholesterol	In vivo	[58]
*Tribulus terrestris* extract	*Tribulus terrestris *	TC, HDL, LDL, TG	In vivo	[44]
Ginsenosides	*Panax spp. *	PPARs, total cholesterol, triglyceride	In vivo	[59]
*Ocimum basilicum *	*Ocimum basilicum *	Total cholesterol, triglycerides, LDL-cholesterol, HDL-cholesterol	In vivo	[60]

**Table 3:** Chinese herbal compounds with antioxidation activity.

Compound	Herb	Target or indicator	Type of study	Reference
*Arisaema tortuosum* tuber extract	*Arisaema tortuosum* Schott	*β*-Glucuronidase; FRAP	In vitro	[61]
Andrographolide derivatives	Andrographolide	VLDL-C, LDL-C, HDL-C; superoxide anions, hydroxyl radicals	In vivo	[62]
Farrerol	*Rhododendron dauricum* L. (ManShanHong)	SOD, GSH-Px; caspase-3, p38 MAPK, Bcl-2	In vitro	[63]
*Celastrus orbiculatus* Thunb.	*Celastrus orbiculatus* Thunb.	TC, non-HDL, TG, apoB100, apoE, HDL; LDL receptor, SR-B1, CYP7A1, HMGCR, CRP, MDA	In vivo	[54]
Tanshinone IIA	*Salvia miltiorrhiza* Bunge	ROS, Bax/Bcl-2, caspase-3, LOX-1, NF-*κ*B, oxLDL, monocyte adhesion, VSMC migration and proliferation, macrophage cholesterol accumulation, TNF-*α*, TGF-*β*1, platelet aggregation, GPx	Both	[64–67]
Cryptotanshinone	*Salvia miltiorrhiza* Bunge	oxLDL, NO, ICAM-1, VCAM-1; monocyte adhesion	In vitro	[42]
Ethanolic fraction of *T. arjuna *	*Terminalia arjuna *	LDL, TG, VLDL, HDL	In vivo	[57]
*Salvia miltiorrhiza* aqueous extract	*Salvia miltiorrhiza* Bunge	Hcy, ROS; PKC/MAPK	In vivo	[68]
*Chlorophytum borivilianum* root extract	*Chlorophytum borivilianum *	LDL oxidation, lipid hydroperoxides, thiobarbituric acid	In vitro	[69]
Aqueous extract of *Buddleja officinalis *	*Buddleja officinalis *	VCAM-1, ICAM-1; ROS; NF-*κ*B	In vitro	[43]
Salvianolic acid B	*Salvia miltiorrhiza* Bunge	oxLDL, ROS, COX, ERK1/2, JNK, MAPK; prostaglandin E2, NADPH oxidase, MMP-2, MMP-9	Both	[70–73]
Caffeoylquinic acids (CQs)	Chwinamul	ROS	Both	[74]
*Epimedium* (Berberidaceae)	*Epimedium spp. *	ROS	Both	[75]
Goji	*Lycium barbarum* and *L. chinense *	SOD, MDA; JNK	Both	[76]
*Ginkgo biloba* extract	*Ginkgo biloba *	VCAM-1, ICAM-1, E-selectin; ROS, RSTF	Both	[46]
*Salvia miltiorrhiza *	*Salvia miltiorrhiza* Bunge	eNOS, NO, NADPH oxidase subunit Nox4	In vitro	[48]
*Scutellaria baicalensis* Georgi flavonoids	*Scutellaria baicalensis* Georgi	SOD	Both	[77]
Emodin	*Rheum rhabarbarum *	ApoE, PPAR-*γ*, GM-CSF, MMP-9	In vivo	[78]
Bisacurone	*Curcuma longa * Linne (Zingiberaceae)	VCAM-1, NF-*κ*B p65, Akt, PKC	In vitro	[49]
*Phyllanthus emblica* extract	*Phyllanthus emblica *	ox-LDL, MDA	In vitro	[79]
Ethanol extract of *Glossogyne tenuifolia *	*Glossogyne tenuifolia *	oxLDL, ROS	In vitro	[80]
*Ocimum basilicum *	*Ocimum basilicum *	total cholesterol, triglycerides, LDL, HDL	In vivo	[60]
Paeonol	*Paeonia lactiflora* Pallas	ICAM-1, NF-*κ*B p65 translocation, ERK, p38	In vitro	[81]
Water extracts of Achyrocline satureoides	*Achyrocline satureoides *	LDL oxidation	In vitro	[82]
Alaternin	*Cassia tora *	NO, Peroxynitrite	In vitro	[83]
Aqueous extract of *Salvia miltiorrhiza *	*Salvia miltiorrhiza* Bunge	Hcy	In vitro	[84]
Gypenosides	*Gynostemma pentaphyllum *	mitochondrial enzyme	In vitro	[85]
Saponins				
baicalein, baicalin and wogonin	*Scutellaria baicalensis *	VSMC proliferation	In vitro	[86]
Scoparone	*Artemisia scoparia *	monocyte adhesion, lipid laden foam cells	In vivo	[87]
Trilinolein	*Panax pseudoginseng *	OFR	In vitro	[88]
Celastrol	*Tripterygium wilfordii* Hook F.	oxLDL, LOX-1, ROS, iNOS, NO, TNF-a, IL-6	In vivo	[89]
Phenolic Rye (*Secale cereale* L.)	Ferulic acid	oxLDL	In vitro	[90]

**Table 4:** Chinese herbal compounds that suppress monocyte migration and activation.

Compound	Herb	Target or indicator	Type of study	Reference
*Prunella vulgaris* ethanol extract	*Prunella vulgaris *	VCAM-1, ICAM-1, E-selectin, ROS; p38 MAPK, ERK	In vitro	[91]
Curcumin	*Curcuma longa *	Macrophage morphological changes	In vitro	[92]
*Panaxnotoginseng *	*Panax notoginseng* saponins	ICAM-1 and VCAM-1	In vivo	[45]
*Ginkgo biloba* extract	*Ginkgo biloba *	VCAM-1, ICAM-1, E-selectin; ROS, RSTF	Both	[46]
*Phyllanthus emblica* extract	*Phyllanthus emblica *	oxLDL, MDA	In vitro	[79]

**Table 5:** Chinese herbal compounds that suppress VSMC migration and proliferation.

Compound	Herb	Target or indicator	Type of study	Reference
Protocatechuic aldehyde	*Salvia miltiorrhiza* Bunge	PI3K/Akt, MAPK, cyclin D2	In vitro	[93]
*Gleditsia sinensis* thorn extract	*Gleditsia sinensis* thorns	MMP-9; p21WAF1, cyclinB1, Cdc2 and Cdc25c; ERK1/2, p38 MAPK, JNK; NF-*κ*B, AP-1	In vitro	[94]
Corynoxeine	Hook of *Uncaria rhynchophylla *	DNA synthesis of VSMCs, ERK1/2	In vivo	[95]
*Phyllanthus emblica* extract	*Phyllanthus emblica *	ox-LDL, MDA	In vitro	[79]
Berberine	*Coptis chinensis *	MAPK1/2, ERK, Egr-1, PDGF, c-Fos, Cyclin D1	In vitro	[96]
Nucifera leaf extract	*Nelumbo nucifera* GAERTN	JNK, MAPK, FAK/PI 3-kinase/small G protein	In vitro	[97]
*Hibiscus sabdariffa* Extract	*Hibiscus sabdariffa* L.	triglyceride, LDL, foam cell formation, VSMC migration	In vivo	[98]
*Panax notoginseng* saponins	*Panax notoginseng *	integrin, FAK, NF-*κ*B	In vivo	[99]
*Astragalus* polysaccharide	*Astragalus membranaceus *	ABCA1, NF-*κ*B	In vitro	[100]
Scoparone	*Artemisia scoparia *	monocyte adhesion, lipid laden foam cells	In vivo	[87]
*Hibiscus sabdariffa* Extract	*Hibiscus sabdariffa* L.	TC, LDL-C; foam cell formation, VSMC migration	In vivo	[98]

**Table 6:** Chinese herbal compounds that suppress foam cell formation.

Compound	Herb	Target or indicator	Type of study	Reference
*Panax notoginseng* saponins	*Panax notoginseng *	integrin, FAK, NF-*κ*B	In vivo	[99]
*Astragalus* polysaccharide	*Astragalus membranaceus *	ABCA1, NF-*κ*B	In vitro	[100]
Scoparone (6,7-dimethoxycoumarin)	*Artemisia scoparia *	monocyte adhesion, lipid laden foam cells	In vivo	[87]
*Hibiscus sabdariffa* Extract	*Hibiscus sabdariffa* L.	TC, LDL-C; foam cell formation, VSMC migration	In vivo	[98]

**Table 7:** Anti-inflammatory Chinese herbal compounds.

Compound	Herb	Target or indicator	Type of study	Reference
*Plectranthus zeylanicus* extracts	*Plectranthus zeylanicus* Benth	5-LO	In vitro	[101]
*Arisaema tortuosum* tuber extract	*Arisaema tortuosum* Schott	*β*-Glucuronidase; FRAP	In vitro	[61]
*Prunella vulgaris* ethanol extract	*Prunella vulgaris *	VCAM-1, ICAM-1, E-selectin, ROS; p38 MAPK, ERK	In vitro	[91]
*Celastrus orbiculatus* Thunb.	*Celastrus orbiculatus* Thunb.	TC, non-HDL, TG, apoB100, apoE, HDL; LDL receptor, SR-B1, CYP7A1, HMGCR, CRP, MDA	In vivo	[54]
2,3,5,4′-Tetrahydroxystilbene-2-O-*β*-D-glucoside (TSG)	*Polygonum multiflorum *	HSP 70, lipocortin 1, Apo A-I; calreticulin, vimentin;	In vivo	[102]
Salvianolic acid B	*Salvia miltiorrhiza* Bunge	JAK2 (Tyr 1007/1008), STAT1 (Tyr701 and Ser727); CXC chemokines' IP-10, Mig, I-TAC; monocyte adhesion; PIAS1, SOCS1	In vitro	[103]
*Cynanchum wilfordii *	*Cynanchum wilfordii *	LDL, HDL, NO, E-selectin, VCAM-1, ICAM-1, ET-1	In vivo	[40]
*Panax notoginseng* extract	*Panax notoginseng *	TNF-*α*, IL-6, TGF-*β*, IL-1*β*	In vivo	[104]
Cryptotanshinone	*Salvia miltiorrhiza* Bunge	oxLDL, NO, ICAM-1, VCAM-1; monocyte adhesion	In vitro	[42]
Salvianolic acid B	*Salvia miltiorrhiza* Bunge	CD40, CD86, CD1a, HLA-DR; IL-12, IL-10, TNF-*α*; TLR4; PPAR*γ*; p38-MAPK, PAI-1, JNK, NF-*κ*B, COX, ERK1/2, prostaglandin E2, NADPH oxidase, MMP-2, MMP-9, oxLDL, ICAM-1, E-selectin	Both	[53, 71, 72, 105, 106]
Tanshinone IIA	*Salvia miltiorrhiza* Bunge	oxLDL, monocyte adhesion, VSMC migration and proliferation, macrophage cholesterol accumulation, TNF-*α*, TGF-*β*1, platelet aggregation, GPx	Both	[66, 67]
Aqueous extract of *Buddleja officinalis *	*Buddleja officinalis *	VCAM-1, ICAM-1; ROS; NF-*κ*B	In vitro	[43]
Artemisinin	*Artemisia annua *	TNF-*α*, IL-1ß, IL-6; NF-*κ*B, IKK*α*/ß, I*κ*B*α*	In vitro	[107]
Evodiamine	*Evodia rutaecarpa *	COX-2, iNOS, prostaglandin E2; HIF-1a; Akt, p70S6K, 4E-BP	In vitro	[108]
*Panax notoginseng *	*Panax notoginseng* saponins	ICAM-1, VCAM-1	In vivo	[45]
Goji	*Lycium barbarum and L. chinense *	SOD, MDA; JNK	Both	[76]
*Ginkgo biloba* extract	*Ginkgo biloba *	VCAM-1, ICAM-1, E-selectin; ROS, RSTF	Both	[46]
*Scutellaria baicalensis *	*Scutellaria baicalensis *	SOD	Both	[77]
Georgi flavonoids	Georgi			
Emodin	*Rheum rhabarbarum *	ApoE, PPAR-*γ*, GM-CSF, MMP-9	In vivo	[78]
Bisacurone	*Curcuma longa* Linne (Zingiberaceae)	VCAM-1, NF-*κ*B p65, Akt, PKC	In vitro	[49]
Feverfew extract	*Tanacetum parthenium *	TNF-*α*, CCL2	In vitro	[109]
Magnolol	*Magnolia officinalis *	IL-6, STAT3, Tyr705 and Ser727, ICAM-1, IREs, monocyte adhesion, cyclin D1, MCP-1	In vitro	[50]
Paeonol	*Paeonia lactiflora* Pallas	ICAM-1, NF-kB p65 translocation, ERK, p38	In vitro	[81]
Aqueous extract of *Salvia miltiorrhiza *	*Salvia miltiorrhiza* Bunge	ICAM-1, VCAM-1, GSH, NF-kB	In vitro	[52]
Magnolol	*Magnolia officinalis *	MCP-1, NF-*κ*B, VCAM-1	In vivo	[51]
*Ginkgo biloba* extract	*Ginkgo biloba *	VCAM-1, ICAM-1	In vitro	[47]
Scoparone	*Artemisia scoparia *	monocyte adhesion, lipid laden foam cells	In vivo	[87]
Celastrol	*Tripterygium wilfordii* Hook F.	oxLDL, LOX-1, ROS, iNOS, NO, TNF-*α*, IL-6	In vivo	[89]

## Abbreviations

5-LO: 5-Lipoxygenase
ABCA1: ATP-binding cassette transporter A1
AP-1: Activator protein-1
ApoB100: Apolipoprotein B100
ApoE: Apolipoprotein E
Cav-1: Caveolin-1
CDC6: Cell division cycle 6
COX: Cyclooxygenase
CRP: C-Reactive protein
CYP7A1: Cholesterol 7*α*-hydroxylase A1
Egr-1: Early growth response protein 1
ERK: Extracellular-signal-regulated kinase
E-selectin: Endothelial cell selectin
ET-1: Endothelin-1
FRAP: Ferric reducing antioxidant power
GM-CSF: Granulocyte-macrophage colony-stimulating factor
GPx: Glutathione peroxidase
GSH: Intracellular glutathione
Hcy: Homocysteine
HGL: High density lipoprotein
HIF-1*α*: Hypoxia-inducible factor 1alpha
HMGCR: 3-Hydroxy-3-methyl-glutaryl-CoA reductase
ICAM-1: Intercellular cell adhesion molecule-1
IL: Interleukin
iNOS: Inducible nitric oxide synthase
IREs: IL-6 response elements
JNK: c-Jun NH2-terminal kinase
KDR, or VEGF receptor-2: Kinase insert domain receptor
LDL: Low density lipoprotein
LOX-1: Oxidized low density lipoprotein receptor-1
MAPK: p38 mitogen-activated protein kinase
MCP-1: Monocyte chemotactic protein-1
MDA: Malondialdehyde
mLDL: Modified low density lipoprotein
MMP: Matrix metalloproteinase
NF-*κ*B: Nuclear factor kappa B
NO: Nitric oxide
non-HDL: Non-high-density lipoprotein
OFR: Oxygen-derived free radicals
oxLDL: Oxidized low-density lipoprotein
PAI-1: Plasminogen activator inhibitor type 1
PDGF: Platelet-derived growth factor
PI3K: Phosphatidylinositol 3-kinase
PPARs: Peroxisome proliferator-activated receptors
ROS: Reactive oxygen species
RSTF: Redox-sensitive transcription factor
SOD: Superoxide dismutase
SR-B1: Scavenger receptor class B type 1
STAT3: Signal transducer and activator of transcription protein 3
TC: Total cholesterol
TG: Triglycerides
TNF-*α*: Tumor necrosis factor-*α*
VCAM-1: Vascular cell adhesion molecule-1
VEGF: Vascular endothelial growth factor
VLDL: Very low density lipoprotein
VSMC: Vascular smooth muscle cell.
